# The Association between Maternal Education Level and Infant Mortality Rates by Gestational Age among Black, White, and Hispanic Infants

**DOI:** 10.1055/a-2717-3838

**Published:** 2025-10-16

**Authors:** J. Camille Goldman, Mykaela J. Watt, Sarah G. Trulove, Rachel Tindal, Vivek V. Shukla, Waldemar A. Carlo, Colm P. Travers

**Affiliations:** 1 Heersink School of Medicine, University of Alabama at Birmingham, Birmingham, Alabama, United States; 2 San Antonio Uniformed Services Health Education Consortium, San Antonio, Texas, United States; 3 Division of Neonatology, Department of Pediatrics, University of Alabama at Birmingham, Birmingham, Alabama, United States

**Keywords:** infant, ethnicity, gestational age, infant mortality, educational status

## Abstract

**Objective:**

This study aimed to determine if maternal educational attainment is associated with racial/ethnic disparities in gestational age (GA)-specific infant mortality rate (IMR).

**Study Design:**

Cohort study using data from the Centers for Disease Control and Prevention (CDC) WONDER expanded linked birth and infant death records database, 2017 to 2019. We included hospital-born infants from 22 to 41 weeks' gestation without major congenital anomalies. We compared GA-specific IMR by maternal race/ethnicity (Black, Hispanic, or White) and maternal education level.

**Results:**

There were 9,356,130 eligible infants during the study period; 16.1% Black, 25.7% Hispanic, and 58.2% White. Black infants born at 23 and 24 weeks' gestation had lower IMR at all maternal education levels than White infants. Black infants from 36 to 40 weeks' gestation had higher IMR except at the lowest maternal education level. Hispanic infants born at 23 and 24 weeks' gestation had lower IMR than White infants across all education levels. At 36 to 40 weeks' gestation, Hispanic infants also had lower IMR except at the highest education level.

**Conclusion:**

The association of maternal educational attainment on GA-specific IMR among late preterm and term infants differs by race and ethnicity. Disparities in IMR persist among Black infants despite educational attainment, while Hispanic infants had lower IMR at lower levels of educational attainment compared with White infants.

**Key Points:**

- The protective effect of maternal education on infant outcomes may vary by race/ethnicity.
- Black late preterm and term infants have higher GA-specific IMRs than White infants, even with similar education levels.
- Hispanic late preterm and term infants show lower mortality rates than White infants at lower education levels.

## Introduction

Infant mortality rate (IMR) is an important indicator of the overall health of a population. A study examining data from 180 countries showed a strong linear relationship between IMR and disability-adjusted life expectancy. 1 Since 1995, the IMR has had a downward trend in the United States and fell by 15% from 2005 to 2017. 2 Despite the fall in total IMR over the years, a difference in IMR between Black and White infants persists. 3 Studies of the U.S. IMR rates show that the relative gap between IMR for Black infants versus White infants has grown. Between 1916 and 2017, the IMR among White infants was reduced by 3.1% each year, but the IMR among Black infants declined by 2.6% annually. 4 This discrepancy in IMR reduction had a significant impact on the Black–White IMR ratio. In 1916, the IMR for Black infants was 87% higher than the rate for White infants, while in 2017, the Black IMR was 122% higher than the White IMR. In the United States, the proportion of Black births in a state is strongly associated with the state's overall IMR. This highlights the significant impact that racial disparities can have at the state level. 5

It is known that race and ethnicity are social constructs, not biological variables, which serve as a proxy for differential exposure to social and structural conditions that influence health, including unequal access to health-promoting resources. 6 7 Along with race, maternal education level has been shown to have an independent association with pregnancy outcomes. The Fundamental Cause Theory proposes that preventative women's health screening was consistently related to educational level, and women with higher education levels were screened more often when compared with their lower education counterparts. 8 Maternal education is one of the largest socioeconomic factors associated with disparities in preterm birth rates between Black and White infants in the United States, accounting for approximately 11.3% of the difference. 9 However, while higher maternal educational attainment is associated with fewer preterm births among White women, higher educational attainment may not be associated with fewer preterm births among Black women, suggesting differential effects of education by race. 10 However, these studies have not assessed the association of maternal education and race with IMR.

The purpose of this study was to analyze the relationship between maternal race and maternal education to determine if there was a significant association between gestational age (GA)-specific IMRs as well as IMRs at a population-level by GA. 5 11 We hypothesized that the disparities in IMR among Black and Hispanic infants compared with White infants occur at each specific GA from 27 to 41 weeks' gestation, despite the level of maternal education attained. 3 Alternatively, disparities in IMR by maternal education and race may be related to differences in the GA distribution, and this may be more accurately reflected using the fetus-at-risk approach. We hypothesized that the disparities in IMR would be present across all GAs using the fetus-at-risk approach. 5 11

## Materials and Methods

### Cohort Selection

The participants in this study are infants registered in the Centers for Disease Prevention and Control (CDC) WONDER expanded linked birth and infant death records database. Deidentified data were obtained from the CDC WONDER database in agreement with the data use restrictions, which limit reporting of subnational data or numbers of infants with less than 10 events. We extracted data from the database using computer code (R version 4.0) to define the inclusion and exclusion criteria and ensure reproducibility. The analysis included live-born infants from 2017 to 2019. We included the following weeks of gestation: extremely preterm (22–27 weeks), very preterm (28–31 weeks), late preterm (32–36 weeks), and term (37–41 weeks).

### Exposure

We included infants whose maternal race was recorded as non-Hispanic Black, non-Hispanic White, or Hispanic in the database, with a known maternal education status, and a maternal age greater than or equal to 20 to allow sufficient years of age for higher levels of educational attainment. Given that non-Hispanic Whites constitute 58.4% of the United States population, Hispanics 19.5%, and Blacks 13.7%, this study focuses on these groups, which together represent approximately 92% of the national population. 12 We excluded infants with major congenital anomalies as well as infants whose cause of death included congenital malformations, deformations, and chromosomal anomalies. The study was approved as not human subjects research by our Institutional Review Board. Strengthening the Reporting of Observational Studies in Epidemiology guidelines were used. 13

### Outcomes

This cohort study compared IMR by maternal race and educational level at each GA. The primary outcome measure was the GA-specific IMR comparing infants with maternal race of non-Hispanic Black with non-Hispanic White. Secondary outcome measures included the GA-specific IMR comparing infants with maternal ethnicity Hispanic, including both Black and White Hispanic infants, with non-Hispanic White. We also compared the population-adjusted IMR at each GA by maternal race/ethnicity and education to account for differences in GA distribution. We calculated the population-adjusted IMR based on the “fetus-at-risk” calculation by subtracting infants already delivered at lower GAs to determine the denominator for subsequent GAs. 5 11 Maternal education levels were categorized based on the mother's highest level of education, and they are defined by the four following groups: no high school diploma or general educational development (GED) testing; high school graduate or GED completed; some college credit but not a degree; and college graduate (including associate degree, bachelor's degree, master's degree, or doctorate).

### Statistical Analysis

We used the sample of all infants who met the inclusion criteria for this population-based study. For each GA and education level, data were compared by maternal race of non-Hispanic Black, non-Hispanic White, or Hispanic to determine IMR, defined as the number of deaths within the first year after birth per 1,000 live-born infants. We calculated relative risk and 95% confidence intervals using White infants as the reference category. Forest plots were created for each comparison, displaying the point estimate and 95% confidence intervals. Findings with a *p* < 0.05 were considered statistically significant. Differences in IMR are presented without adjustment for known confounders to illustrate the unmitigated burden of disparities, as controlling for these factors would potentially mask the structural determinants underlying these inequities.

### Ethics Approval

This study used deidentified patient data from a publicly available database, and patient consent was not required. The study was approved as not human subjects research by our Institutional Review Board.

## Results

There were 9,356,130 infants that were born during the study period; 1,504,230 (16.1%) Black infants; 2,406,327 (25.7%) Hispanic infants; and 5,445,573 (58.2%) White infants. There were higher rates of college graduation among mothers of White infants compared with mothers of Black or Hispanic infants ( Table 1 ). There were higher rates of mothers whose highest level of education was either a high school diploma or GED among mothers of Black and Hispanic infants compared with White infants. Mothers of Hispanic infants had a higher rate of not completing high school compared with mothers of Black and White infants.

**Table 1:** Highest maternal education achieved by race/ethnicity

Level of education	White; *n* = 5,445,573	Black; *n* = 1,504,230	Hispanic; *n* = 2,406,327
College graduate, *n* (%)	2,948,949 (54.1)	410,763 (27.3)	537,037 (22.3)
Some college, no degree, *n* (%)	1,079,485 (19.8)	404,008 (26.9)	502,518 (20.9)
High school graduate or GED completed, *n* (%)	1,124,727 (20.7)	528,154 (35.1)	781,531 (32.5)
12th grade or less with no diploma, *n* (%)	292,412 (5.4)	161,305 (10.7)	585,241 (24.3)

Abbreviation: GED, general educational development.

**Fig. 1:**
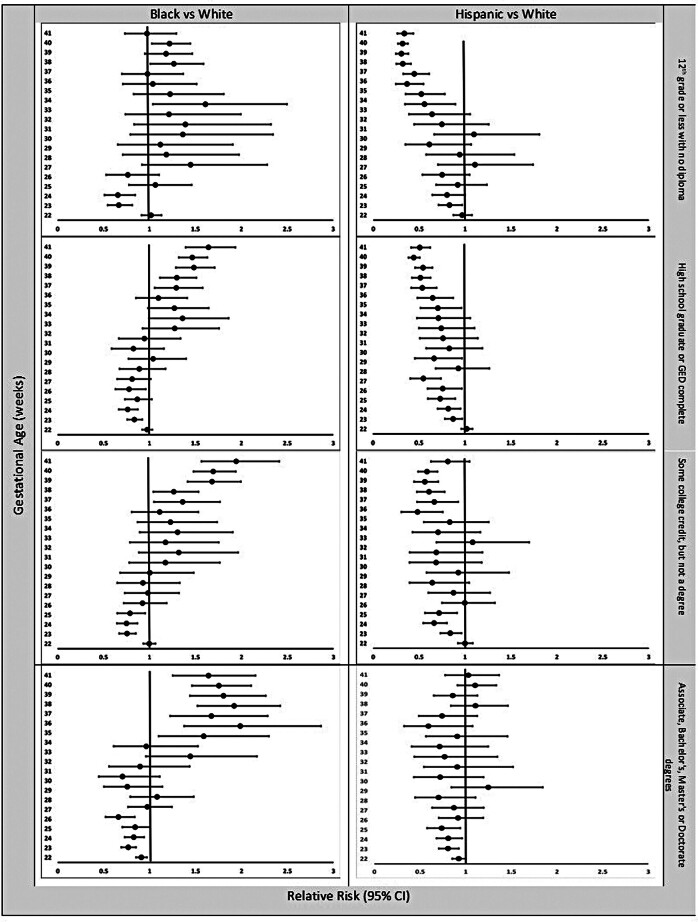
Relative risk and 95% confidence intervals for the gestational age-specific infant mortality rate (IMR) from 41 to 22 weeks by race/ethnicity. Black and Hispanic infants are compared with White Infants subdivided by maternal educational level.

Among college graduates, Black term infants (37–41 weeks' gestation) had a higher gestational-age-specific IMR, whereas extremely preterm infants at 23 and 24 weeks' gestation had a lower IMR compared with White infants ( Fig. 1 ; Table 2 ). Similar patterns were seen when comparing Black Infants and White infants with mothers who had completed some college without receiving a degree, as well as among those with a high school diploma or GED ( Fig. 1 ). For Black mothers without a high school diploma or GED, infants born at 23 and 24 weeks' also had lower IMRs compared with infants born to White mothers, while those born at 34, 38, and 40 weeks' had higher IMRs ( Fig. 1 ; Table 2 ).

**Table 2:** Gestational age-specific infant mortality rates (IMR) subdivided by race and highest educational level

GA	Black				White				Hispanic			
	12th grade or less with no diploma	High school graduate or GED completed	Some college credit, but no degree	Associate, bachelor's, master's, or doctorate degree	12th grade or less with no diploma	High school graduate or GED completed	Some college credit, but no degree	Associate, bachelor's, master's, or doctorate degree	12th grade or less with no diploma	High school graduate or GED completed	Some college credit, but not a degree	Associate, bachelor's, master's, or doctorate degree
22	838.9	809.1	817.6	762.4	825.4	830.3	820.4	838.7	799.1	846.8	820.3	774.2
23	388.2	472.5	418.6	431.2	582.9	565.3	554.2	561.8	483.8	492.6	465.2	453.1
24	236.2	260.1	260.4	285.2	363.3	341.1	347.5	345.1	291.6	279.1	230.3	279.0
25	211.3	189.0	168.4	163.7	198.5	217.9	213.9	194.4	182.6	159.3	153.7	143.2
26	110.1	108.9	108.0	83.1	144.0	140.0	116.9	126.0	108.0	106.3	116.6	115.5
27	94.2	86.0	67.7	77.6	65.1	106.1	68.9	79.7	72.0	58.1	60.3	69.4
28	54.8	50.3	39.2	42.5	46.3	56.5	42.3	39.3	43.6	52.5	27.2	27.6
29	43.1	44.6	32.7	22.0	38.5	43.0	32.6	29.1	23.5	28.5	30.2	36.3
30	36.7	24.1	23.9	14.2	27.0	29.2	20.4	20.1	29.6	24.2	14.0	14.5
31	30.7	19.0	21.4	11.3	22.1	20.2	16.3	12.6	16.5	15.4	11.2	11.4
32	20.8	17.8	13.3	11.1	17.1	14.0	11.3	7.7	11.0	10.4	12.3	5.9
33	23.1	13.5	11.4	5.8	14.4	9.9	8.7	6.0	8.0	7.1	6.2	4.3
34	13.2	10.2	6.9	5.2	10.8	8.0	5.6	3.3	5.7	5.6	4.7	3.0
35	9.3	7.2	5.6	4.2	9.0	6.6	5.1	2.1	3.3	4.3	2.5	1.3
36	6.2	6.1	4.6	2.8	6.4	4.8	3.4	1.7	2.8	2.6	2.3	1.2
37	6.6	4.4	3.3	2.0	5.2	3.4	2.6	1.0	1.7	1.7	1.6	1.1
38	4.7	3.5	3.1	1.4	4.0	2.4	1.8	0.7	1.2	1.3	1.0	0.6
39	3.9	3.0	2.3	1.0	3.2	2.0	1.3	0.6	1.0	0.9	0.8	0.6
40	2.8	2.8	2.0	0.9	2.9	1.7	1.1	0.5	1.0	0.9	0.9	0.5
41	1.8	2.2	2.2	0.8	2.0	1.1	1.1	0.6	0.8	1.1	0.9	0.4

Abbreviations: GA, gestational age; GED, general educational development.

For infants born to Hispanic mothers, there was a gestational-age-specific survival advantage seen across all educational levels among infants at the lowest GAs of 23 and 24 weeks, as well as some other extremely preterm GAs at different levels of education ( Fig. 1 ; Table 2 ). At the lowest level of education, Hispanic infants born from 34 weeks' gestation or later had lower IMR at each GA when compared with White infants. Patterns were similar among those whose mother's highest level of education was either a High school diploma or completing some college, but did not include all GAs from 34 weeks or higher. Among Hispanic infants whose parents had any college degree, there was no difference in gestational-age-specific IMR from 26 weeks' gestation or higher.

**Fig. 2:**
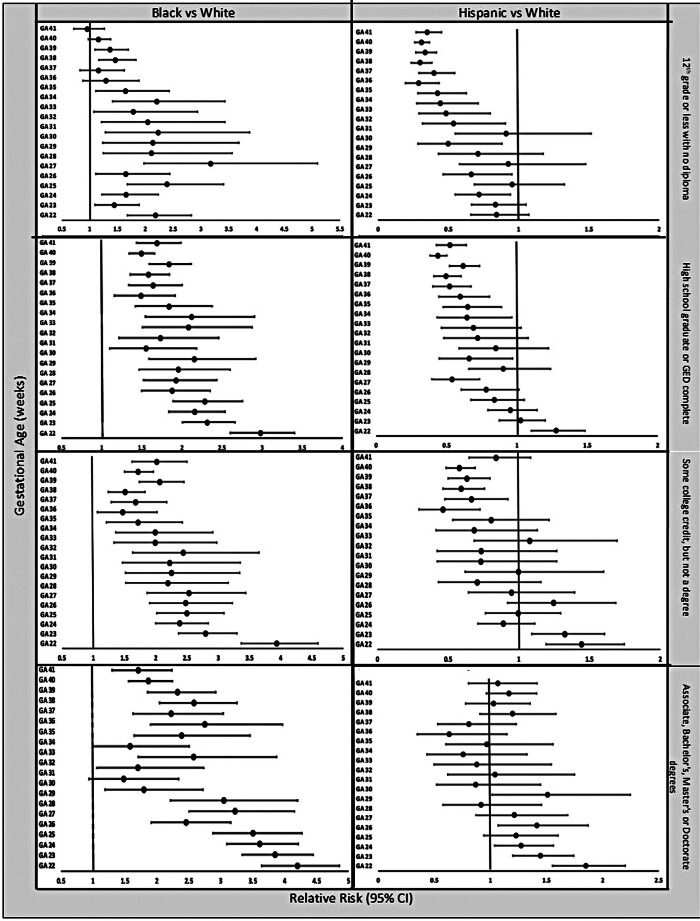
Relative risk and 95% confidence intervals for the gestational age-specific infant mortality rate (IMR) from 41 to 22 weeks by race/ethnicity adjusted for the population-at-risk. Black and Hispanic infants are compared with White Infants subdivided by maternal educational level.

When adjusted for population-at-risk, Black infants had a higher risk of infant mortality at all GAs among those whose mothers completed their high-school education and those with some college when compared with White infants whose mothers had the same level of education ( Fig. 2 ). At the highest level of educational attainment, there were significant differences at most but not all GAs. Among those at the lowest level of educational attainment, there were differences in survival at lower GAs but not among term infants and some late preterm groups. Differences were largest among those at the lowest gestations, reflecting the higher rate of extremely and very preterm birth among Black infants in the United States.

Hispanic infants at the lowest GAs had a higher risk of IMR based on the population-at-risk analyses, suggesting a higher risk of preterm birth at the lowest gestations affecting the Hispanic populations with high-school or higher education levels compared with White infants. However, at higher GAs, the results for the population-at-risk analyses comparing Hispanic with White infants did not differ substantially from the results for the GA-specific IMR analyses across educational groups.

## Discussion

This population-based study found that the association of educational attainment with IMR varies by race/ethnicity. The disparities in IMR among term Black and White infants are less pronounced at the lowest education levels compared with higher education levels. Paradoxically, the lower IMR favoring Hispanic infants at lower levels of education is no longer present at the highest education levels. Furthermore, the lower GA-specific IMR among Black and Hispanic infants at the lowest gestations was reversed when analyzed based on the fetus-at-risk approach. 5 11 These data suggest that the relatively small survival benefit among individual Black and Hispanic infants born at the lowest gestations is more than offset by the higher number of births, and therefore, deaths at a population level.

Whereas studies examining disparities at a population level report worsening disparities in IMR, studies examining outcomes among extremely preterm infants found either decreasing disparities or no disparities in care practices, major morbidities, and mortality. 14 15 However, these studies adjusted for multiple baseline clinical risk factors, including GA, birth weight, sex, and multiple births, and maternal demographic differences, including maternal education, marital status, and insurance, between groups. Furthermore, it has been noted that comparing outcomes among preterm birth cohorts can lead to bias depending on whether data are analyzed by group or by fetus-at-risk to account for the entire population. 11 This, in turn, may explain the “so-called” preterm infant paradox, 16 where Black extremely preterm infants have been reported to have a survival advantage at an individual level compared with White infants but have higher rates of infant mortality at each GA when analyzed at a population level. 3 While Black infants born extremely preterm experience higher rates of mortality, there is evidence that clinical severity alone does not fully account for these disparities. A recent cohort study found that maternal race and ethnicity were associated with a lower likelihood of redirection-of-care discussions and actions, independent of education and insurance status. 17 These differences may reflect variation in cultural values, religious beliefs, understanding of prognosis, or clinician bias. Such findings suggest that observed disparities in extremely preterm infant outcomes may stem from unequal access to or engagement in end-of-life decision-making processes.

Our study agrees with data from the 2007 to 2008 NCHS Cohort Linked Live Birth—Infant Death Files, which found that the risk of overall infant mortality was lower for Hispanic infants when compared with non-Hispanic White and non-Hispanic Black infants, respectively. 18 Several studies have also shown that infants born to Hispanic mothers appear to have a paradoxical advantage when it comes to infant health and mortality when compared with their non-Hispanic counterparts. 19 20 In our study, Hispanic infants had a lower risk of death, which was most pronounced at lower levels of educational attainment, while there were limited differences at the highest levels of educational attainment. This is in contrast to the higher risk of death among Black infants at higher levels of educational attainment, with more limited differences among those at the lowest levels of educational attainment.

There are known differences in IMR within racial/ethnic groups by level of educational attainment, suggesting that higher levels of education may be protective. In California, from 2007 to 2015, mothers with a high school education or less accounted for 48.5% of the total births but 59% of infant deaths. 21 College education or higher was associated with lower IMR. Furthermore, infants of Black mothers with the lowest level of education had the highest risk of mortality compared with White infants or infants of Black mothers with college-education or higher (both *p* < 0.001). However, Black infants of college-educated or higher mothers still had higher infant mortality than White infants of college-educated or higher mothers ( *p* < 0.001). These differences are in line with our study, which showed that college education is not sufficient to fully attenuate the racial disparities in IMR among Black infants in the United States.

Our study analyzed national data, but results from individual states in the United States may differ. A 15-year U.S. study that followed the IMR rates of each state found that only 13 of the 50 states made a statistically significant reduction in the Black–White IMR ratio. The rest of the states showed that while both Black and White IMR rates were decreasing, the disparity in the Black–White IMR ratio was sustained from 2000 to 2012. 22 In a study from Wisconsin, the IMR among Black infants born to mothers with high-school education or less was higher than those of White mothers or Black mothers with higher levels of education, but this study did not examine results by GA which is the largest determinant of IMR. 23 Older U.S. data from 1998 to 2002 reported an educational gradient in IMR with greater protection from higher education for White infants relative to Black infants. 24 In contrast, infants born to Hispanic mothers may have similar IMR when compared with infants of White Mothers after adjustment for maternal education. 25 Taken together with our investigation, current data suggest that while education is protective against IMR, the effect size varies by race/ethnicity.

Low birth weight, birth, and prematurity account for much of the higher IMR in the United States. 26 There is also an association between maternal education and low birth weight births. A meta-analysis found that high levels of maternal education are associated with a 33% lower risk of low birth weight. 27 In a study from the 1980s using a national database to examine infant outcomes, infants of Black mothers with college education were more than twice as likely to have low birth weight births when compared with infants with White college-educated mothers. 28 Furthermore, there was no difference in preterm birth rates among the most socially disadvantaged and lowest-educated Black and White mothers. Similar results have been reported in a study using higher levels of education in a model of socioeconomic status to examine the association between race and preterm birth. 29 It has also been reported that the protective association of maternal education on preterm birth may be decreasing over time. 30 In this study, the differences in the population-level fetus-at-risk analyses suggested that an important difference in preterm birth rates drives overall disparities in IMR.

The persistent disparities in GA-specific IMRs among late preterm and term Black infants, even after considering educational attainment, have been the subject of extensive research. The “weathering hypothesis” proposes that chronic stress experienced throughout a Black individual's life may negatively impact reproductive health, contributing to higher rates of prematurity and infant mortality within this population. 31 Stress can impact biological aging, and Black women had telomere lengths consistent with being approximately 7.5 years older than White women. 32 Furthermore, maternal mortality is associated with higher rates of infant mortality. A study examining the relationship between maternal and infant health found that countries that made progress in reducing maternal mortality also experienced improvements in infant and child mortality rates during the same period. 33 Black women are more than three times more likely to die during the postnatal period compared with their White counterparts, highlighting significant racial disparities in maternal health outcomes. 34 The risk of maternal death rises with increasing BMI, and Black women have disproportionately high obesity rates even at higher levels of income, whereas higher income was protective against obesity in White and Hispanic women. 35 36 The racial disparity in pregnancy-related morbidity and mortality may contribute to the persistently higher rates of infant mortality among Black infants. These data suggest that the disparity in Black infant mortality reflects a complex, multifactorial mix of social determinants of health that are not mitigated by maternal educational attainment.

The Hispanic Paradox, characterized by better health outcomes and lower IMRs among Hispanic immigrants in the United States despite socioeconomic disadvantages, has been subject to various explanatory hypotheses. One of these is immigration selectivity, positing that Hispanic immigrants may be positively selective on good health, with healthier individuals more likely to migrate than those with poorer health. This selectivity results in a higher concentration of healthy individuals within the immigrant population, potentially contributing to improved health outcomes. 37 38 Furthermore, researchers have studied cultural patterns, which suggest that certain cultural aspects within Hispanic communities promote healthy behaviors and strong family ties, contributing to relatively favorable health and mortality patterns. 37 Our study found that as Hispanic mothers achieved higher levels of education, the rate of infant mortality was not lower compared with White infants. Our fetus-at-risk analysis suggests that Hispanic infants born to mothers with higher education are at higher risk of preterm birth. These findings may be explained by the negative impact of acculturation, the process by which individuals or groups adopt and incorporate elements of a new culture into their own culture. It occurs when individuals from one cultural background come into continuous contact with another culture and gradually adopt aspects of that culture while retaining some aspects of their original cultural identity. As immigrants and their descendants become more acculturated, they may be more prone to adopt less healthy behaviors. 39 Studies exploring acculturation and maternal health behaviors found that mothers with a higher degree of U.S. cultural adaptation are more likely to adopt unhealthy behaviors such as smoking during pregnancy, potentially contributing to a decline in health outcomes. 40 41 Analysis of the Latino National survey showed that educational attainment can predict English Mastery, a core acculturation marker, even after correcting for generational standing and income, suggesting that higher education may coincide with greater acculturation. 42 43

## Limitations

In this population-based study, we did not adjust for differences in baseline characteristics, including pregnancy complications between groups, due to database restrictions on access to individual patient data. The large sample size and the matching for GA in our comparison groups may have reduced the risk of confounding, but remaining biases may have impacted the estimated effect sizes reported in this study. We did not have access to data on the day of birth within each week category, and it is possible this differed by race/ethnicity. We did not have data on fetal loss, and previous studies have reported higher fetal mortality rates impacting Black women, such that our study may underestimate racial/ethnic differences using the fetus-at-risk approach. 44 In addition, redirection of care practices may differ among infants by race/ethnicity, but these data were not available. 17 We did not adjust for multiple testing, and given the large number of comparisons, it is possible that some results may have been significant or not significant by chance. This study compared differences across maternal education levels but did not examine differences across paternal education levels. We did not consider the association between education and mortality rates among infants born to women of races/ethnicities other than Black, White, and Hispanic mothers, which represent approximately 8% of births in the United States.

## Conclusion

Disparities in GA-specific IMRs among late preterm and term Black infants persist despite educational attainment. Hispanic infants have a lower gestational-age-specific infant mortality at lower levels of educational attainment, consistent with the Hispanic Paradox.
